# The drooling reduction intervention trial (DRI): a single blind trial comparing the efficacy of glycopyrronium and hyoscine on drooling in children with neurodisability

**DOI:** 10.1186/1745-6215-15-60

**Published:** 2014-02-17

**Authors:** Jeremy R Parr, Emma Weldon, Lindsay Pennington, Nick Steen, Jane Williams, Charlie Fairhurst, Anne O’Hare, Raj Lodh, Allan Colver

**Affiliations:** 1 Institute of Neuroscience, Newcastle University, 3rd Floor Sir James Spence Institute, Royal Victoria Infirmary, Newcastle upon Tyne NE1 4LP, UK; 2 Institute of Health and Society, Newcastle University, Newcastle upon Tyne, UK; 3 Nottingham University Hospital, Nottingham, UK; 4 Department of Paediatric Neurosciences, Evelina Children’s Hospital, London, UK; 5 School of Clinical Science, University of Edinburgh, Edinburgh, UK; 6 Great North Children’s Hospital, Newcastle upon Tyne, UK

**Keywords:** Randomised trial, Drooling, Neurodisability, Glycopyrronium, Hyoscine, Children, Treatment satisfaction, Drooling impact scale

## Abstract

**Background:**

Drooling saliva is a common problem in children with neurodevelopmental disorders. The negative consequences of drooling include skin breakdown, dehydration, and damage to clothing and equipment. Children and families often suffer social embarrassment due to drooling. There is no evidence about the relative effectiveness, side effect profiles or patient acceptability of the two medications most commonly used to reduce drooling - glycopyrronium and hyoscine. Consequently, there is no consensus or guideline to aid clinical decisions about which drug to use, and at what dose.

**Methods/design:**

A multi-centre, randomised trial of treatment with glycopyrronium or hyoscine in children with problematic drooling and non-progressive neurodisability. Ninety children aged between 3 and 15 years who have never received medication for drooling will be stratified by severity of drooling and care centre. Randomisation to receive treatment with glycopyrronium or hyoscine will be computer generated from the trial randomisation website. Dose adjustment and side effect monitoring will occur via telephone consultation. Medication arm will be known to participants and clinicians but not the Trial Outcome Assessor.

The primary outcome measure is the Drooling Impact Scale score at four weeks, at which time all children will be on the maximum tolerated dose of their medication. Secondary outcome measures include change in Drooling Impact Scale score between baseline, 4, 12 and 52 weeks, change in Drooling Severity and Frequency Scale score and difference between groups in the Treatment Satisfaction Questionnaire for Medication score. A structured interview with children and young people of sufficient age, cognitive and communication ability will explore their perceptions of drooling and the effectiveness and acceptability of the medications.

**Discussion:**

The primary objective of the study is to identify whether glycopyrronium or hyoscine is more effective in treating drooling in children with non-progressive neurodisability. The study will also determine which medications at what doses are most acceptable and have fewest side effects. This information will be used to develop evidence based guidance to inform the medical treatment of drooling.

**DRI trial registration:**

Current Controlled Trials: ISRCTN75287237.

EUDRACT: 2013-000863-94.

Medicines and Healthcare products Regulatory Agency (MHRA): 17136/0264/001-0003.

## Background

### Study rationale

Drooling saliva, due to oro-motor impairment, is a common problem in children with neurodevelopmental disorders. The negative consequences of drooling for children and their relatives include skin breakdown of the child’s chin, dehydration, and damage to clothing and equipment such as electronic communication aids; furthermore, children, their siblings and parents often suffer social embarrassment due to the physical appearance of drooling and salivary spray when talking and eating [1]. Parents may have to remind children to swallow pooled saliva. Bibs, or wristbands are used to soak up saliva and clothing changes are frequently required, resulting in extra washing loads [1,2]. There is no evidence about the relative effectiveness of the medications most commonly used to reduce drooling. There are only limited data about the medications’ side effects and how acceptable they are to children and parents; this lack of information makes it difficult for children, parents and doctors to make informed decisions about which drug to use, and at what dose [1].

Around 35% of children with cerebral palsy have problematic drooling, equating to around 10,500 children in the UK [3-5]. Children with cerebral palsy with Gross Motor Classification System (GMFCS) levels IV and V are five and thirteen times respectively more likely to experience problematic drooling than children classified as GMFCS level I [3], and Parkes, personal communication. A survey we conducted showed that UK paediatricians see on average one treatment naive child per month with problematic drooling [6].

### Current treatment and its limitations

Medication is the first intervention used to treat drooling by most UK clinicians. Both glycopyrronium and hyoscine work by reducing cholinergic stimulation of salivary glands, and the volume of saliva produced. As many other bodily functions are under cholinergic control, medications have adverse effects such as constipation, urinary retention and difficulties in hot weather due to reduced sweating. Blurred vision, sedation, irritability and increased seizure frequency are possible neurological effects [1]. There has never been a study to investigate how different drug dosages should be increased to achieve optimal balance between drug effectiveness and adverse effects, nor which drug achieves the better balance [1]. In our survey [6], 98% of the 151 paediatricians surveyed reported using medication for the treatment of problematic drooling: 85% used hyoscine first line and glycopyrronium second. Ninety-four percent of paediatricians asked children and parents about the effectiveness of medication; just over half also asked other professionals. Only seven of the 151 paediatricians used a scale to monitor effectiveness. Paediatricians generally considered that hyoscine led to more adverse effects than glycopyrronium; hyoscine was also associated with more frequent dose reduction and withdrawal of medication [6].

Other interventions used to ameliorate drooling include [1]:

● Oro-motor exercises and postural management. There is little evidence that they alone improve drooling

● Intra-oral devices. These are dangerous in children with limited tongue movement, or epilepsy - each commonly seen in children with neurodisability

● Surgical excision of or re-routing of salivary glands. This can reduce drooling, but it is an invasive procedure, and can be associated with negative effects due to a lack of saliva, such as difficulty in chewing

● Botulinum toxin injection into the salivary glands has shown benefits. However, injections (for which a general anaesthetic is usually required) are needed every six to twelve months. Botulinum is only available through a few UK clinical services, and therefore, few children can benefit

## Methods and design

### Aim

The aim of the study is to determine the clinical effectiveness and adverse effect profile of glycopyrronium and hyoscine in children with problematic drooling.

### Primary objective

To identify whether glycopyrronium or hyoscine is more effective in treating drooling in children with non-progressive disability.

### Secondary objectives

To determine the most effective dosing schedule to achieve optimum balance between clinical benefits and adverse effects and to describe the reported satisfaction with medication treatment of drooling for children and carers.

### Primary outcome measure

The primary outcome measure is the Drooling Impact Scale (DIS) [7] score at four weeks, by which time all children will be on the maximum tolerated dose of their medication.

### Secondary outcome measures

● Change in DIS score between baseline, 4, 12 and 52 weeks

● Change in Drooling Severity and Frequency Scale (DSFS) score [8] at 4, 12 and 52 weeks

● Difference between the groups in the Treatment Satisfaction Questionnaire for Medication (TSQM-version II) score [9] at 4, 12 and 52 weeks

● Final medication doses, as a percentage of child’s maximum dose, compared between the two groups

● Relationship between dose and adverse effects, investigated using survival analysis

● Children and young people’s perceptions of the effectiveness and acceptability of the medications, elicited in structured interviews

### Study design

The study is a multi-centre (15 sites), randomised trial of two commonly used medications, glycopyrronium and hyoscine, in children with problematic drooling. Medication type will be known to parent, child and clinicians but not the Trial Outcome Assessor (TOA). Ninety children, who have never received medication for drooling, will be recruited.

### Subject population

The trial will recruit children with non-progressive neurodisability who require treatment for drooling. This includes children with any neurodevelopmental disorder, such as cerebral palsy or Down syndrome, where the primary cause of disability is non-progressive (the motor and functional disability of children may change as they age but the primary cause of the neurodisability does not worsen). Children with progressive disability will not be included in the study as their oro-motor impairment may worsen over time, making interpretation of response to medication difficult.

### Inclusion criteria

Treatment naive children, with non-progressive neurodisability, who require glycopyrronium or hyoscine to reduce drooling

● No contra-indication to either medication

● Age ≥ 36 months to under 16 years at the start of trial medication

● Weight ≥ 10 kg

### Exclusion criteria

● Children who have received medical or surgical interventions for drooling

● Children with medical conditions for which either medication is contraindicated

● Children whose parents are considered unable to follow the study protocol

● Parents without a mobile or home telephone

● Parents who cannot complete a telephone call in English

● Children already in another clinical trial that involves trial medication that could affect drooling, or drooling management

● Pregnancy

● Previous withdrawal from this study

### Identification and recruitment of participants

Recruitment of participants is by the local paediatrician during standard clinical care. The paediatrician explains the study and gives the parent written information; when appropriate, written information is also given to the child.

We offer three recruitment approaches to suit the family and local paediatrician:

1. If the parent has no doubts about their child taking part in the trial, they can sign the consent form straight away. The local paediatrician randomises the patient to a treatment arm using the online randomisation system. The family can then collect the medication from the hospital pharmacy. It is unusual to seek consent straight away but for the reasons that follow, we received permission from the Research Ethics Committee (REC) to allow this:or

● The trial takes place in the context of routine clinical practice and the medicines are already being used

● It is more convenient for families to collect the medication from the hospital straight away. Otherwise the family, who might have travelled considerable distances to attend, would have to travel back to the hospital to pick up the medication

● The medication does not start on the day of consent. The Trial Research Paediatrician (TRP) phones the family the next day to ensure that the family has no doubts or any further questions about trial participation.

2. Families, whose paediatrician may be in a community clinic without web access, can sign the consent form and the family goes home without medication. The TRP or local paediatrician then does the randomisation as soon as possible, and then phones the family who collect the medication from the hospital, or it is sent to them by secure pharmacy courier.

or

3. The family may want to think further about whether they want their child to join the trial. If subsequently they want to participate, approach 1 or 2 above would be adopted.

### Consent procedures

The local paediatrician takes written consent from parents. Assent is sought from children deemed able to give meaningful assent by their parents and the local paediatrician.

The original signed consent form is retained in the Investigator site file, with a copy in the clinical notes and a copy provided to the participant. The participant consents to their family doctor being informed of their participation in the study. In addition, they are asked to consent to the person who gives medication in school to be informed of their child’s participation in the trial, and for that person to contact the TRP if necessary.

For eligible families who do not want to take part in the trial, we will record the reasons given for nonparticipation. The study has ethical approval to collect the following data from non-participants to enable investigation of representativeness of the study families: child’s neurodisability diagnosis, sex, year of birth, and parent’s postcode (to enable an assessment of social bias).

### Randomization and blinding

Stratification is by two criteria:

● Severity of salivation over the previous week using the modified Mier classification [10]:

● Saliva usually only on lips or chin

● Saliva on lips, chin and clothes

● Clinical centre

Randomization is via a password-protected web-based service provided by the Newcastle Clinical Trials Unit. Patients are allocated to one of the two therapies in the ratio 1:1 using random permuted blocks of variable length. The sequence is revealed after data lock. Randomisation occurs after recruitment, at least 24 hours before start of medication.

### Intervention

The medication regime is:

1. Glycopyrronium arm

Week 1: 40mcg/kg/per dose

Week 2: 60mcg/kg/per dose

Week 3: 80mcg/kg/per dose

Week 4: 100mcg/kg/per dose

All doses three times a day, adjusted according to response up to maximum 2 mg per dose.

The medication is given by syringe orally or by a child’s feeding tube.

2. Hyoscine arm

Week 1: ¼ patch

Week 2: ½ patch

Week 3: ¾ patch

Week 4: full patch

The patch is placed below the ear and is replaced every three days, alternating sites to minimise the risk of a local skin reaction. The backing of the patch is cut to expose the prescribed portion of the patch; the patch itself is not cut because it is a reservoir patch that would leak product. An occlusive dressing is then applied over the patch as per usual clinical practice.

In either arm, medication is increased weekly through the trial from weeks 1 to 4; thereafter, or when the maximum tolerated dose is achieved, the participants will remain on that dose. Trial medication then continues for 12 weeks.

### Stages for the family

Participants receive a study medication pack which contains: study medication with labelling; parent information sheet to accompany medication; information sheet regarding telephone calls; and occlusive dressings (when in the hyoscine arm). Within a day of randomisation the family is phoned by the TRP, who confirms continued willingness to be in the trial and addresses any queries. The family then receives weekly phone calls from the TRP and the medication dosage is adjusted up to four weeks. Weekly phone calls continue to six weeks, then fortnightly to twelve weeks with side effect information being actively sought and noted at each call. At 12 weeks, the child exits the trial and care is returned to the local paediatrician at a pre-arranged appointment. Medication, packaging and any remaining medication is returned to local pharmacies for destruction. All families will receive a final phone call nine months later (one year after starting medication) to record their medication type and dose at that date. These stages are summarised in Table 1.

**Table 1:** Trial stages for all families and children

**Time**	**Start**	**Day medication starts**	**1 week after medication starts**	**2 weeks after medication starts**	**3 weeks after medication starts**	**4 weeks after medication starts**	**12 weeks after medication starts**	**52 weeks after medication starts**
Study discussed in clinic	X							
Consent	X							
Randomisation	X							
Local Paediatrician: medication prescribed	X							
Trial Research Paediatrician: medication started		phone call						
Trial Research Paediatrician: medication dose adjustments			phone call	phone call	phone call	phone call	phone call	phone call
Adverse effects assessed								
Trial Outcome Assessor: primary and secondary outcome measures		phone call				phone call	phone call	phone call
remaining medication returned							X	

Within a day of randomisation the family is also phoned by the TOA, who is blind to the treatment arm, to gather baseline data. Each time the TOA contacts the family she reminds them that she should not be informed of the treatment arm the participant is in.

### Side effects and adverse events

Dose dependent, predictable side effects are not regarded as adverse events. These side effects are reviewed at the weekly telephone conversation with the TRP for the first six weeks and fortnightly until week twelve. Parents can contact the TRP to discuss side effects or concerns between 0900 and 1700 during the week; out of usual daytime hours, the parent should contact their family doctor or local child health service. Any participant reporting clinically significant side effects will have their dose decreased to the previous week’s dose, which will then be continued for the duration of the 12 week trial.

True adverse events will be recorded and monitored. Elective or scheduled treatment for pre-existing conditions that did not worsen during the study or any pre-planned hospitalisations not associated with clinical deterioration are not regarded as adverse events. If serious adverse events are reported over the telephone, the participant will be advised to seek local medical attention. If serious adverse events present to local services for management (whether or not related to the investigational medication), they will be brought to the attention of the Chief Investigator (CI) immediately and he shall determine seriousness and causality in conjunction with involved medical practitioners. The CI and Trial Manager are responsible for reporting all treatment-related serious adverse events to the REC and Trial Sponsor within the relevant time frames.

### Interviews with young people

The TRP will visit a subset of families whose children have sufficient age, cognitive and communication ability to report their opinion about their drooling and treatment in a structured interview. When necessary, children will be supported by their parents or local professionals to use any usual communication aids. Interviews will take place in home or at school. The aim of these interviews is to set the primary and secondary outcomes of the study in the context of a child’s experience. Some example questions are shown in Table 2.

**Table 2:** Sample questions for the structured interview of a subset of families and children

You are taking medicine for your drooling - it is the patch behind your ear or the medicine you take	
Do you think the medicine worked?	yes/no/not sure
What was your drooling like before you took the medicine?	very bad/bad/OK/good/very good
What is your drooling like now?	very bad/bad/OK/good/very good
Has the medicine helped you?	yes/no/not sure
If yes, how has it helped you? Symbols for face not sore, dry chin, dry clothes, friends, happy.	
Have you felt unwell/poorly (word used by family) whilst taking the medicine?	yes/no/not sure
If unwell:	
Which part of you has felt unwell? Cartoon of a child, child points to relevant body parts or interviewer points to each part of the body.	
How unwell have you felt?	OK/bad/very bad
Did you feel unwell like that before you took the medicine?	yes/no/not sure
Did you want to stop taking the medicine?	yes/no/not sure
Are you still feeling unwell?	yes/no/not sure
Did you tell your mum/dad about this?	yes/no/not sure
If no: ‘I think I should tell them and we can talk about how to help you get better’	
If a friend needed help for their drooling, should they take the medicine you have tried?	yes/no/not sure

Children will be asked to indicate their response to questions verbally or by pointing/eye pointing to visual symbols printed above written words in the response sets below.

### Concomitant medication and treatment

Other medication and treatment is prescribed or administered as required by local health providers in usual clinical care; details are recorded in the case report form (CRF).

### Researcher skills

The TRP is a paediatric registrar, EW, with a specialist interest in community paediatrics. She has six years’ experience in paediatric medicine, including one year in community paediatrics and three months in paediatric neurology. When absent for any reason, her role will be covered by JP or AC, both consultant paediatricians.

The TOA has had training in the use of the outcome tools, including pilot telephone calls with non-participants.

### Data collection, storage and record keeping

The local paediatrician provides the TRP and TOA with the patient’s name, address, postcode and telephone number via the secure National Health Service (NHS) Email system or secure fax. He/she also provides data about main neurodisability diagnosis and other relevant clinical features. The online randomisation system generates a unique study number that identifies the patient; this number is used by central trial staff when discussing the patient with the local paediatrician. Assessments collected by the TOA and drug dosage, side effects and adverse event data collected by the TRP are entered on paper, then transferred to a database, with a Good Clinical Practice (GCP) [11] compliant electronic data capture system. In order for a link to be made with the patient name and the study number, a log is kept in the Investigator site file, and by the central trial team. All patient identifiable information is kept in a locked cabinet, with access limited to authorised members of the research team. All study data are retained and handled in accordance with GCP [11] and local policy and in accordance with the Data Protection Act 1998. Documentation of prescribing, dispensing and return of study medication is maintained for study records. The final trial dataset will be initially available only to the study team; after the results of the trial have been reported, anonymous trial data will be made publicly available.

### Sample size calculation

The primary outcome measure is the DIS (range 0 to 100), which has a standard deviation of 13 [7]. The study is powered to detect a clinically significant difference of 10.0 in the mean score between groups, representing what parents viewed as the difference between ‘very good to excellent’ and ‘good’ in a global rating. To detect this mean difference of 10.0 with 90% power, assuming a type 1 error rate of 5%, data from two groups of 36 are required. Allowing for 20% loss to follow up or attrition, recruitment and randomisation of 90 children is needed. A final sample size of 72 gives 90% power to detect an effect size of 0.77 in the secondary outcome measures. For the TSQM, this corresponds to mean differences of 18.2, 17.6, 13.2 and 16.5 in the effectiveness, side effects, convenience and overall satisfaction subscales respectively.

### Statistical analysis

Data will be analysed by the trial statistician using SPSS software (Armonk, NY, USA).

For the primary outcome analysis, the DIS score at four weeks will be compared between the two groups using an independent sample *t*-test.

The secondary outcomes will be analysed as follows:

● Change in DIS score between baseline, 4 weeks, 12 weeks and 52 weeks; we will use repeated measures analysis of variance to look at changes in the DIS over time comparing the two groups

● Change in DSFS score; we will use repeated measures analysis of variance to look at changes in the DSFS over time comparing the two groups

● Difference between the groups in the TSQM score; we will use repeated measures analysis of variance to look at changes in the TSQM over time comparing the two groups

● The final medication doses, as a percentage of child’s maximum dose, will be compared between the two groups with the exact Mann–Whitney test

● The relationship between dose and adverse effects will be investigated using survival analysis. The dependent variable will be the dose corresponding to a side effect that was deemed to be ‘not tolerable’. Observations are censored if (i) maximum dose is reached with no side effects or (ii) a decision is made not to increase the dose for any other reason. The two groups will then be compared using a Cox proportional hazards model. Results will be given with a 95% confidence interval for the hazard ratio.

The impact of missing data will be assessed using a sensitivity analysis. Any data imputation will depend upon the level of missing data and its pattern. There will be no interim analysis.

Data from the structured interviews will by synthesised by the TRP in collaboration with the trial statistician. Summary statistics will be mainly descriptive; where appropriate interval estimates of parameters of interest will be generated (for example for the proportion of respondents reporting positive responses to a particular issue).

### Withdrawal of participants

Participants have the right to withdraw from the study at any time for any reason, and without giving a reason. The Investigator also has the right to withdraw patients from the study in the event of inter-current illness, adverse events, serious adverse events, suspected unexpected serious adverse reactions, protocol violations, or administrative reasons. Where families withdraw without starting medication or within the first 48 hours, they will be replaced.

Should a patient or parent decide to withdraw from the study, they will notify the local paediatrician or the TRP. All efforts will be made to report the reason for withdrawal as thoroughly as possible. The TRP will send parents who decide to withdraw a Withdrawal Letter. They will be asked if they agree to data collected prior to their child’s withdrawal from the study being used for the purposes of the trial, and whether they agree to one phone call about what happened with the management of their child’s drooling (at one year after starting the study). If the participant and family wish to withdraw completely then none of their data will be analysed.

### Discontinuation rules

The trial may be prematurely discontinued on the basis of new safety information, or for other reasons given by the Data Monitoring and Ethics Committee (DMEC), Trial Steering Committee (TSC), Sponsor, regulatory authority or ethics committee (see below). For individual participants, the study drug will be discontinued if there are adverse effects that warrant this, or the participant requests it.

### Management of the trial, quality control and assurance

The trial is managed through Newcastle Clinical Trials Unit (CTU) and the research team’s Trial Management Group (TMG).

The Principal Investigators at the recruiting sites are responsible for the day-to-day study conduct at their sites. Newcastle CTU provides day-to-day support for the sites and provides training through Investigator meetings, site initiation phone calls and routine monitoring. Quality control is maintained through adherence to the study protocol, study specific working instructions, the principles of GCP [11], research governance and clinical trial regulations.

An independent DMEC consisting of independent chair, two physicians not connected to the trial, and one statistician, will provide independent review. Its purpose is to monitor efficacy and safety endpoints. The DMEC will have access to unblinded study data. The committee will have discussions (not necessarily face to face) at least three times: around the start of the trial, six months into recruitment and at the end of the study. At the first meeting, the DMEC will discuss and advise on the inclusion of an interim analysis at five months, and possible adoption of a formal stopping rule for efficacy or safety.

A TSC with an independent chair will provide overall supervision of the trial. The TSC will meet every six months during the trial. Its role is to monitor progress and supervise the trial to ensure it is conducted to high standards in accordance with the protocol, the principles of GCP, relevant regulations and guidelines with regard to participant safety.

The Trial Manager from the CTU will ensure that the study is conducted in accordance with GCP. The Trial Manager’s main areas of focus will include consent, serious adverse events, essential documents in study files and drug accountability. Monitoring of study processes will have two components:

1. Central monitoring of each recruiting site will involve:

● Checking inclusion/exclusion criteria and consent processes

● Version verification of consent form for 100% of patients

● Confirmation via checklist of all essential documentation being present in the site file; including regulatory approvals and amendments

● Checking eligibility data for 10% of participants entered in the study

● Checking drug accountability and management

2. Central monitoring of data collected by the TRP and TOA at Newcastle will start soon after the first participant has been recruited, and will then be undertaken again six months into recruitment. Monitoring will involve verification of 10% of reported serious adverse events against source data, and review of primary endpoint data.

### Plans to communicate trial results to participants and the public

The results of the trial will be sent to all participants and will be submitted to a peer reviewed journal for publication. The results will be used to develop evidence based guidance to inform the medical treatment of drooling.

### Protocol publication

This protocol has been written according to and in adherence with recommendations from the SPIRIT checklist: recommended items to address in a clinical trial protocol and related documents [12].

### Ethics committee and regulatory approval

The study will be conducted in accordance with ethical principles as listed in the Declaration of Helsinki [13]. Ethics approval was granted by Newcastle and North Tyneside1 REC on 10 May 2013, number 13/NE/0078. A first substantial amendment was approved on 23 July 2013. A second substantial amendment was approved on 17 September 2013 before commencing the trial recruitment on 24 October 2013. (Trial Protocol version 2.0, 19/8/13).

If further protocol amendments are needed, approval will be sought from the Sponsor and then submitted to the REC.

## Discussion

This study will determine whether there is a difference in the effectiveness of the two trial medications, and which doses are associated with least side effects. This information will be used to develop evidence-based guidance to help children, parents and doctors: firstly to decide which medication to prescribe to reduce problematic drooling and at what dose; secondly to show how adverse effects can be monitored, and when dosage should be reduced or medication stopped.

The study will benefit children with neurodisability who drool, and their families. By identifying how best to choose medication, decide on dosage and monitor effect and side effects, this applied research relates to the day to day practice of health service staff - specifically, paediatricians and speech and language therapists. Children’s drooling will be better controlled with the accompanying physical and psychosocial benefits for child and family.

## Trial status

The trial is in progress at a number of sites and started to recruit in October 2013.

## Abbreviations

CI: Chief Investigator; CRF: case report form; CTU: Clinical Trials Unit; DIS: Drooling Impact Scale; DMEC: Data Monitoring and Ethics Committee; DSFS: Drooling Severity and Frequency Scale; GCP: Good Clinical Practice; GMFCS: Gross Motor Classification System; MHRA: Medicines and Healthcare products Regulatory Agency; REC: Research Ethics Committee; TMG: Trial Management Group; TOA: Trial Outcome Assessor; TRP: Trial Research Paediatrician; TSC: Trial Steering Committee; TSQM: Treatment Satisfaction Questionnaire for Medication.

## Competing interests

The authors declare that they have no competing interests.

## Authors’ contributions

JP is the Chief Investigator. JP and AC wrote the original protocol. JP, AC and EW have written and revised the manuscript for submission. JP, AC and LP, JW, CF and AOH were part of the team that conceived and planned the trial. LP, NS, JW, CF, AOH and RL contributed to the development of the protocol. All authors read and approved the final manuscript.
